# Advancing Human iPSC-Derived Cardiomyocyte Hypoxia Resistance for Cardiac Regenerative Therapies through a Systematic Assessment of In Vitro Conditioning

**DOI:** 10.3390/ijms25179627

**Published:** 2024-09-05

**Authors:** Caroline A. Snyder, Kiera D. Dwyer, Kareen L. K. Coulombe

**Affiliations:** Institute for Biology, Engineering and Medicine, School of Engineering, Brown University, Providence, RI 02912, USA; caroline_a_snyder@alumni.brown.edu (C.A.S.); kiera_dwyer@brown.edu (K.D.D.)

**Keywords:** human induced pluripotent stem cell derived cardiomyocytes (hiPSC-CMs), tissue engineering, engineered cardiac tissue (ECTs), myocardial infarction (MI), ischemia, hypoxia resistance, cardiac metabolism, heart regeneration

## Abstract

Acute myocardial infarction (MI) is a sudden, severe cardiac ischemic event that results in the death of up to one billion cardiomyocytes (CMs) and subsequent decrease in cardiac function. Engineered cardiac tissues (ECTs) are a promising approach to deliver the necessary mass of CMs to remuscularize the heart. However, the hypoxic environment of the heart post-MI presents a critical challenge for CM engraftment. Here, we present a high-throughput, systematic study targeting several physiological features of human induced pluripotent stem cell-derived CMs (hiPSC-CMs), including metabolism, Wnt signaling, substrate, heat shock, apoptosis, and mitochondrial stabilization, to assess their efficacy in promoting ischemia resistance in hiPSC-CMs. The results of 2D experiments identify hypoxia preconditioning (HPC) and metabolic conditioning as having a significant influence on hiPSC-CM function in normoxia and hypoxia. Within 3D engineered cardiac tissues (ECTs), metabolic conditioning with maturation media (MM), featuring high fatty acid and calcium concentration, results in a 1.5-fold increase in active stress generation as compared to RPMI/B27 control ECTs in normoxic conditions. Yet, this functional improvement is lost after hypoxia treatment. Interestingly, HPC can partially rescue the function of MM-treated ECTs after hypoxia. Our systematic and iterative approach provides a strong foundation for assessing and leveraging in vitro culture conditions to enhance the hypoxia resistance, and thus the successful clinical translation, of hiPSC-CMs in cardiac regenerative therapies.

## 1. Introduction

Cardiovascular disease is the leading cause of death worldwide [1]. Myocardial infarction (MI) is especially devastating, resulting in the loss of blood flow to part of the heart muscle and the ensuing death of up to one billion cardiomyocytes (CMs) [2]. Due to the limited intrinsic regenerative capacity of the heart, MI results in a significant loss of contractile function and puts patients at high risk of heart failure [3,4]. Engineered cardiac tissues (ECTs) represent an emerging regenerative therapy that aims to incorporate functional CMs into the existing muscle to improve the contractile function of the heart. 

Several challenges must be addressed to facilitate the translation of ECTs to the clinic. First, for ECTs to have a relevant therapeutic impact on the adult human heart post-MI, an extremely high dose of cells (one billion CMs) must be delivered [5,6]. Previous work in our lab has shown that scale-up of ECTs has significant effects on tissue structure and function, with higher cell density corresponding to decreased elastic modulus, collagen organization, prestrain development, and active stress generation [7]. In order to achieve the necessary cell dose for therapeutic benefit while maintaining mechanical integrity of the ECT, it is imperative to maximize CM engraftment, which poses a second challenge. Successful translation of ECTs for heart regeneration requires implanting the ECT into the harsh, often hypoxic environment of the heart post-MI. Following acute MI, the heart undergoes a multiphase repair process characterized by reactive oxygen species (ROS) generation, proinflammatory cytokine production, localized hypoxia, and stiffening of the native tissue over time [8,9]. Hypoxia presents an especially important challenge for ECT engraftment success because even with a prefabricated vascular network or inclusion of proangiogenic proteins, it can take days to weeks to vascularize cell-dense grafts in clinically relevant sizes [10,11]. The combination of localized hypoxia and limited vascularization immediately following ECT implantation necessitates improved ischemia resistance of CMs in ECTs, with the goal of maximizing cell survival, tissue mechanical properties, and therapeutic benefit.

CM maturation is a key factor to consider in the context of ischemia resistance. It has been well established in the literature that human induced pluripotent stem cell (hiPSC)-derived CMs (hiPSC-CMs) in vitro are developmentally immature compared to resident CMs in the adult heart, as measured by their morphology, structural components, electrophysiology, contractile strength, metabolism, and gene expression [12]. A major focus in the field of heart regeneration is the development of methods to improve the maturation of human embryonic stem cell (hESC) or hiPSC-derived CMs, with approaches including metabolic conditioning to activate fatty acid (FA) oxidation, a hallmark of adult CMs [13]; electrical and mechanical conditioning to improve conduction velocity of electrical signals and contractile force generation [14]; and nanotopological substrate cues to promote sarcomere alignment within CMs [15]. However, each approach is limited in scope, often addressing or analyzing a single facet of cell maturation to improve a narrowly defined functional outcome. Particularly in the context of cardiac regenerative medicine, the ideal CM maturation state has yet to be identified. In fact, recent work highlights that fully mature hESC/hiPSC-CMs may not be well suited to implantation in a post-MI heart, as more mature CM phenotypes have a greater oxygen demand that cannot be met in the hypoxic, post-MI environment [16,17]. In addition to modulating CM maturation state through metabolic, electrical, and mechanical strategies, several other features of CM physiology have been examined in the literature with links to ischemia resistance. Signaling pathways, such as the Wnt signaling pathway, are important for CM differentiation and proliferation (often in the context of development and hiPSC cardiac differentiation) and can be targeted for ischemia resistance [18]. Mitochondrial stabilization [19], apoptosis inhibition [20], and short-term pre-exposure to extreme conditions (such as heat shock [21,22], moderate hypothermia [23,24], and hypoxia preconditioning (HPC) [25,26]) are diverse pathways that we hypothesize may influence hiPSC-CM ischemia resistance.

In this study, a range of factors across several aspects of cell physiology, including metabolic conditioning, Wnt signaling, culture substrate, alignment, electrical stimulation, heat shock, hypoxia preconditioning (HPC), mitochondrial stabilization, and apoptosis inhibition, were selected based on consensus in the literature as key targets for modulating hiPSC-CMs response to hypoxia through distinct pathways. In terms of metabolic conditioning, it has been well documented that immature CMs primarily use glycolysis, instead of the more efficient but machinery-intensive oxidative phosphorylation of mature adult CMs [12]. Such metabolic pathways are important in terms of hypoxia resistance of CMs, as oxidative phosphorylation, despite promoting maturation in terms of metabolism, contraction, and electrophysiology, has an extremely high oxygen demand that may not be well met in a post-MI heart [13,27,28,29,30]. The ability to balance both metabolic machinery maturation and hypoxia resistance is explored through treating hiPSC-CMs with metabolic substrates to promote oxidative phosphorylation (i.e., fatty acids), glycolysis (i.e., high glucose), or both. Modulation of the Wnt pathway is also considered, as Wnt activation has been shown to maintain contractile generation and kinetics of hiPSC-CMs while shifting gene expression to that of a more immature phenotype [18], yet its role in hypoxia has not yet been thoroughly explored. Additionally, in vitro environmental conditions that promote specific aspects of CM maturation are explored to determine their unique impacts on hiPSC-CM response to hypoxia. For example, soft substrate culture, which is used to tune CM stiffness toward that of the myocardium, is tested as it has previously been demonstrated to improve hiPSC-CM engraftment [15] as well as influence CM function in terms of contractile stress generation and calcium handling [31]. Alignment cues, which physically guide CMs for improved sarcomere alignment and thus greater contractile stress generation [32,33], as well as electrical stimulation, which improves CM mechanical, structural, and electrical maturation [34], are also tested, as their impact on CM hypoxia susceptibility or resistance has not yet been explored. Next, heat shock and HPC are utilized, as they are able to initiate complex cascades of compensatory signaling to maintain CM survival in response to higher temperature through the expression of heat shock proteins, which reduce apoptosis through mechanisms such as macromolecule stabilization, protein folding guidance, and misfolded protein removal [22,35], as well as in response to hypoxia through the expression of potent transcriptional factors such as hypoxia inducible factor 1 (HIF-1) to preserve CM function [36,37,38,39]. Lastly, given the knowledge of hypoxia induced death in CMs, two targets to prevent such death are explored, which include mitochondrial stabilization through the use of a mitochondrial-targeted antioxidant to decrease oxidative stress [19] and apoptosis inhibition by cyclosporine A, which stabilizes the mitochondrial transmembrane potential to prevent the release of cytochrome c [20]. Conditions were screened by assessing cell function of treated and untreated hiPSC-CMs in 2D culture in normoxic (ambient O_2_, 5% CO_2_) and hypoxic (1% O_2_, 5% CO_2_) environments. Select treatments that reduce cellular stress during hypoxia were then applied to 3D engineered cardiac tissues (ECTs) to quantify formation, mechanics, morphology, and functional response to hypoxia. In both 2D and 3D experiments, metabolic conditioning and HPC had significant benefits on cell function and tissue mechanical properties upon exposure to hypoxia. Our work highlights the need to assess advancements in the field through the lens of application in regenerative therapies, and also identifies practical, key criteria to promote the hypoxia resistance of hiPSC-CMs. 

## 2. Results

### 2.1. High-Throughput Assessment of Hypoxia Protective Treatments Using 2D hiPSC-CMs 

**Figure 1:**
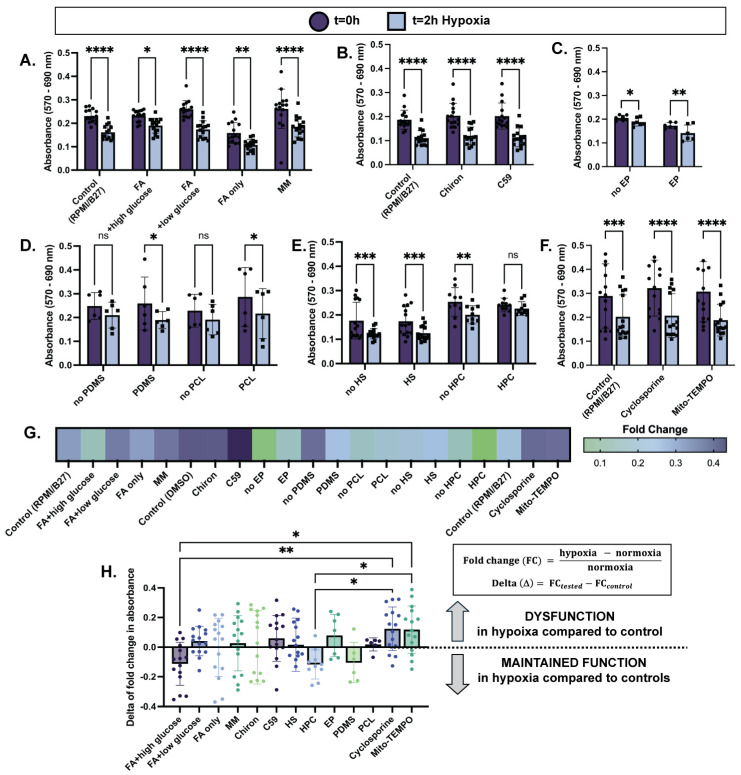
**Cellular dysfunction assessed through metabolic stress with 2 h of hypoxia is reduced in select treatment conditions.** MTT assay results before and after 2 h of hypoxia exposure (1% O_2_, 5% CO_2_) are displayed as absorbance (570 nm–690 nm) of 2D plated hiPSC-CMs treated with (**A**) varying culture media compositions for one week; (**B**) Wnt activation/inhibition overnight; (**C**) electrical pacing (EP) for one week; (**D**) PDMS or PCL substrate for one week; (**E**) heat shock (HS) for one hour 24 h prior to hypoxia, or hypoxia preconditioning (HPC) for 30 min 24 h prior to hypoxia; (**F**) apoptosis inhibitor (Cyclosporine A) or mitochondrial stabilizer (Mito-TEMPO) for 24 h. (**G**) Fold change in cell metabolic activity from normoxia to hypoxia shows less of an impact of hypoxia on viability (i.e., lower fold change) in select treatment groups. (**H**) Delta of fold change in cell metabolic activity is calculated as shown (right) and displayed (left) to compare across treatment conditions. *n =* 6–15 per group, with significance defined as * *p* < 0.05; ** *p* < 0.01; *** *p* < 0.001; **** *p* < 0.0001.

Maintaining cell viability in hypoxia is imperative for cardiac cell-based regenerative therapies, as CMs are implanted into the hypoxic, post-infarct environment. Due to limited revascularization in the infarct zone, implanted cells are subjected to persistent localized hypoxia [9,10] until grafts can be vascularized. We hypothesize that the resilience of hiPSC-CMs to hypoxia that influences cell engraftment and survival can be improved by modulating CM phenotype with specific in vitro priming treatments of the hiPSC-CMs. After an extensive literature search, the following treatment categories were chosen to evaluate their impact on maintaining CM function in hypoxia: cellular metabolism (RPMI/B27; fatty acid [FA] with no, low, or high glucose; maturation media [MM]); Wnt signaling modulation (activation with Chiron, inhibition with C59); electrical pacing; substrate (PDMS, PCL); pre-exposure to extreme conditions (heat shock, hypoxia preconditioning); and drug treatment (cyclosporine A, mito-TEMPO mitochondrial stabilization). Because manipulation of metabolism has been instrumental in the field for maturing hiPSC-CMs in vitro, is widely adopted, and plays a pivotal role in cell stress pathways influencing cell death, the MTT assay was used to assess CM viability based on mitochondrial enzyme activity (of succinate-dehydrogenase) during short periods of hypoxia exposure (Supplemental Figure S2A). After confirming that changes in cell metabolic activity could be detected after only two hours of hypoxia (Supplemental Figure S2B), the MTT assay was used to assess the selected treatment conditions in a high-throughput manner (Supplemental Figure S2C). An overview of the MTT results, with absorbance taken at t = 0 of hypoxia and t = 2 h of hypoxia, are displayed in Figure 1A–F.

In terms of cellular metabolism, after exposure to two hours of hypoxia, hiPSC-CMs cultured in FA + high glucose media showed the smallest decrease in absorbance (0.18 ± 0.04) compared to t = 0 normoxia controls, and therefore the smallest decrease in cell metabolic activity while in hypoxia (Figure 1A,G). Interestingly, high glucose-only media (RPMI/B27) and FA-only media exhibited similar decreases in metabolic activity after hypoxia (0.29 ± 0.04 and 0.29 ± 0.051, respectively), while FA + low glucose media and maturation media (MM) exhibited the greatest decreases in cell metabolic activity (0.33 ± 0.03 and 0.34 ± 0.05, respectively) while in hypoxia (Figure 1A,G). Although comparisons of fold change between groups were not statistically significant, these trends suggest that a combination of FA and glucose availability during hypoxia promotes cell function. Further, modulation of the Wnt signaling pathway, electrical pacing, and the use of an aligned PCL scaffold did not improve cell metabolic activity while in hypoxia compared to their controls (Figure 1B–D,G). However, the use of a PDMS soft substrate slightly improved cell metabolic activity in hypoxia compared to its non-substrate control (0.24 ± 0.06 and 0.35 ± 0.13 decrease in absorbance, respectively) (Figure 1D,G). Pre-exposure to extreme conditions for a short duration prior to damage induction was also evaluated for cardioprotection in hypoxia. While heat shock did not improve hypoxia response, HPC did significantly maintain cell metabolic activity in hypoxia compared to its control (0.057 ± 0.03 and 0.18 ± 0.07, respectively) (Figure 1E,G). This result is especially convincing because the no-HPC and HPC conditions had similar absorbances at t = 0 (0.25 ± 0.02 and 0.24 ± 0.009, respectively), meaning that improved cell metabolic activity viability was likely due to improved hypoxia response as opposed to initial cell death during the preconditioning period. Lastly, drug treatments targeting apoptosis inhibition (Cyclosporine) and mitochondrial stabilization (Mito-TEMPO) resulted in no improvements compared to the control (Figure 1F,G).

The above analyses were made by comparing each experimental group to its own control group (i.e., FA + high glucose media compared to RPMI/B27; PDMS compared to no PDMS; HPC compared to no HPC, etc.). As indicated by smaller decreases in cell metabolic activity compared to the control, FA + high glucose media, PDMS soft substrate, and HPC demonstrated potential beneficial effects on hiPSC-CM response to hypoxia, with the effects of metabolic conditioning and HPC being the most pronounced (Figure 1G). 

To better compare groups across conditions, delta of fold change was calculated by subtracting the mean fold change in the control group from each fold change in the treatment group (i.e., PDMS fold change—mean no PDMS fold change; HPC fold change—mean no HPC fold change, etc.) (Figure 1H). Fold change indicates the decrease in cell metabolic activity from normoxia to hypoxia, with smaller fold changes corresponding to smaller decreases of metabolic activity while hiPSC-CMs are exposed to hypoxia. Thus, a negative value of delta of fold change then indicates that the treatment group had a smaller decrease in cell metabolic activity than the control group, and therefore an improved response to hypoxia. The FA + high glucose cell culture media and HPC treatment groups have the most negative values for delta of fold change (−0.11 ± 0.04 and −0.12 ± 0.03, respectively), suggesting that these treatments have the potential to improve hiPSC-CM ischemia resistance. Other groups have a distribution of positive and negative values. Notably, cell culture media conditions with low glucose content performed poorly (0.04 ± 0.02, FA + low glucose; −0.0019 ± 0.05, FA only; 0.03 ± 0.05, MM), consistent with previous findings that metabolically immature cells performing anaerobic glycolysis as opposed to fatty acid oxidation have greater ischemia resistance [17]. 

Cell death in hypoxia was also quantified in a subset of treatment groups using LIVE/DEAD staining (Supplemental Figure S3A–C). These results revealed that there was little cell death with 6 h of hypoxia exposure (0.54 ± 0.21% dead cells) and only 1.20 ± 0.27% dead cells (*p* < 0.01) with 12 h of hypoxia exposure (Supplemental Figure S3D). These results are consistent with work conducted by other groups [17,40], and such limited cell death during oxygen deprivation (1% O_2_) is promising for the ultimate long-term success of cell engraftment for cell-based regenerative therapies. These results also justify our use of the MTT assay as a preliminary, high-throughput measure of cellular dysfunction prior to complete cell death in prolonged hypoxia. 

### 2.2. ECT Fabrication and Formation with Select Culture Conditions

**Figure 2:**
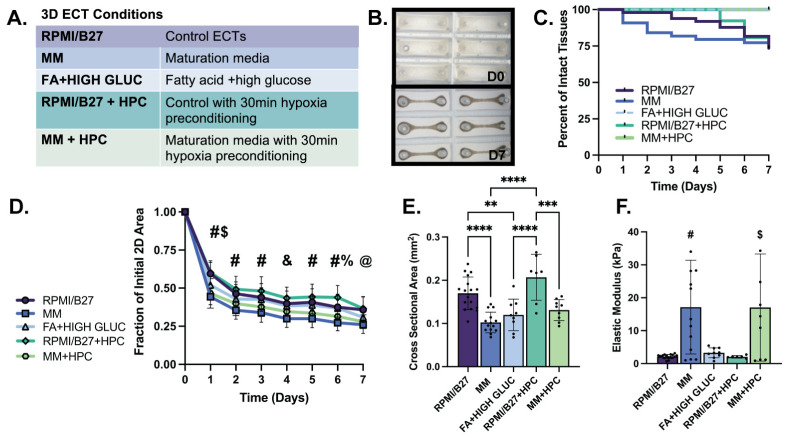
**Formation of ECTs with select conditions is comparable to standard (RPMI/B27) ECTs, with extent of 2D compaction correlating with CSA and elastic modulus.** (**A**) Conditions obtained from 2D screening results used for ECT testing. (**B**) Brightfield images illustrating tissue compaction from initial casting on day 0 (D0) to day 7 (D7) of in vitro culture (mold size, 3 × 9 mm). (**C**) Survival curve showing percentage of intact ECTs to assess structural survival. (**D**) Quantification of tissue compaction over 7-day culture; (**E**) Quantification of tissue cross sectional area at D7 of culture. (**F**) Elastic modulus for ECTs shows functional stiffness of tissues. *n* = 6–17 samples per group, with significance defined as ** *p* < 0.01; *** *p* < 0.001, **** *p* < 0.0001. # represents significance between all groups except RPMI vs. FA; RPMI vs. RPMI-HPC; MM vs. MM-HPC; FA vs. RPMI-HPC; and FA vs. MM-HPC. $ represents significance between RPMI vs. FA and FA vs. RPMI-HPC; & represents significance between RPMI vs. MM; MM vs. FA; MM vs. RPMI-HPC; and RPMI-HPC vs. MM HPC. % represents significance between RPMI vs. RPMI-HPC and FA vs. RPMI-HPC. @ represents significance between RPMI vs. MM; RPMI vs. MM-HPC; MM vs. FA; and RPMI-HPC vs. MM HPC.

The results of 2D cell screening demonstrated that cell culture media with different metabolic substrates and HPC have significant benefits in curbing dysfunction in hypoxia. Specifically, high glucose conditions perform better than low glucose conditions relative to control culture medium (RPMI/B27). Additionally, HPC has a strong positive effect in curbing dysfunction in hypoxia. Based on these results, a spectrum of metabolic conditions (Control RPMI/B27, FA + high glucose, and MM) and HPC conditions (RPMI + HPC and MM + HPC) were selected to evaluate functional endpoints for regenerative therapy applications through the fabrication of 3D ECTs (Figure 2A).

Formation and persistence of ECTs in the various growth conditions was consistent with standard ECT control conditions through one week of culture, as evidenced by ECT compaction and beating uniformity throughout culture (Figure 2B). Structural survival, defined as intact tissues that did not neck and break over seven days of culture, was also consistent across treatment conditions compared to the RPMI/B27 control group (range: 74–100% at day 7) (Figure 2C). All ECT conditions significantly compacted to form a syncytium, with the extent of compaction (tissue area tracked from tissue casting on D0 to D7) quantified throughout culture (Figure 2D). MM-treated ECTs compacted to the smallest fraction of their initial area by D7 in culture (0.26 ± 0.01 in MM group and 0.28 ± 0.01 in MM + HPC group) compared to RPMI/B27 (0.36 ± 0.01), RPMI + HPC (0.46 ± 0.02), and FA + high glucose (0.31 ± 0.01) ECTs. The cross-sectional area (CSA) of the ECTs correlated well with the 2D compaction, as MM-treated ECTs had the smallest CSA (0.10 ± 0.01 mm^2^ in MM and 0.13 ± 0.01 mm^2^ in MM + HPC), followed by the FA + high glucose ECTs (0.12 ± 0.01 mm^2^) and RPMI/B27 ECTs (0.17 ± 0.01 mm^2^ in RPMI/B27 ECTs and 0.21 ± 0.02 mm^2^ in RPMI/B27 + HPC) (Figure 2E). Passive mechanics of the ECTs, as measured through elastic modulus, was highest in the MM-treated ECTs (17.16 ± 3.95 kPa in MM and 17.04 ± 5.43 kPa in MM + HPC), followed by FA + high glucose (3.29 ± 0.50 kPa), RPMI/B27 (2.14 ± 0.16 kPa), and RPMI/B27 + HPC (2.06 ± 0.16 kPa) ECTs (Figure 2F).

Immunohistochemical staining confirmed that there were intact (healthy) nuclei and sarcomeres throughout the thickness of ECTs in all treatment conditions (Supplemental Figure S4A–E). MLC2 isoforms were also compared across conditions, with the ventricular isoform (MLC2v) being predominately expressed across all groups as an indication of sarcomere maturation (vs. the MLC2a isoform developmentally expressed earlier in ventricular lineage CMs; Supplemental Figure S4F–K). Overall, ECT formation and development were most impacted by the metabolic substrates in the culture media, with MM (high FA, low glucose concentrations) significantly increasing ECT compaction and stiffness.

### 2.3. Active Mechanical Properties of ECTs in Normoxia

Cardiac tissue contractile function, crucial to the future therapeutic benefit of ECTs in vivo, was next investigated using isometric tensile mechanical testing (Supplemental Figure S5A) to evaluate the impact of the treatments on normoxic ECT force generation and after ECT exposure to hypoxia (24 h, 1% O_2_, 5% CO_2_).

**Figure 3:**
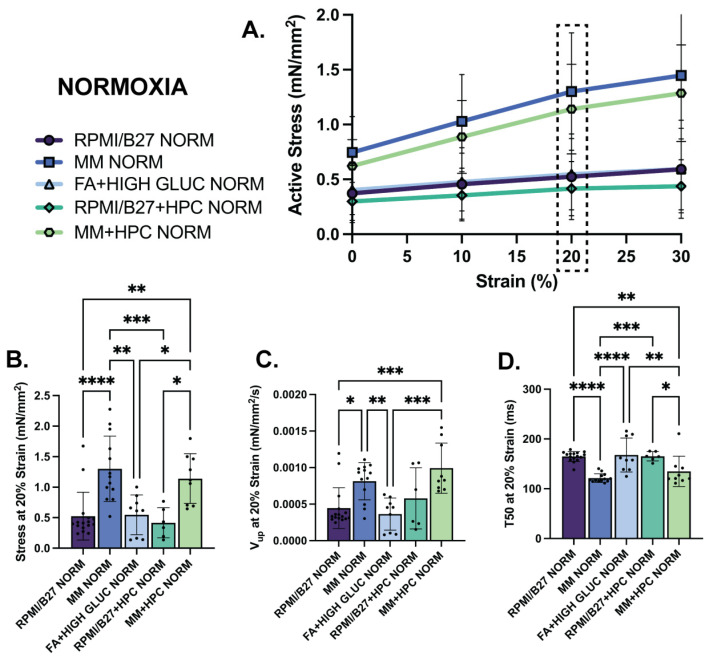
**MM-treated ECTs exhibit enhanced mechanical function assessed through active stress generation and kinetics.** (**A**) Active stress generation of normoxia ECTs from 0 to 30% stretch, measured at increments of 10% strain. (**B**) Active stress generation, (**C**) upstroke velocity (V_up_), and (**D**) time to 50% relaxation (T_50_) at 20% strain. *n* = 6–17 samples per group, with significance defined as * *p* < 0.5; ** *p* < 0.01; *** *p* < 0.001; **** *p* < 0.0001. Dashed box indicates 20% strain, which was the strain used to present the subsequent data.

In normoxia, the MM and MM + HPC groups had the strongest contractile metrics at all levels of prestrain up to 30% of initial resting length (Figure 3A). At the physiological relevant strain of 20%, for example, MM ECTs had the highest active stress generation at 1.30 ± 0.15 mN/mm^2^, followed by MM + HPC ECTs at 1.14 ± 0.14 mN/mm^2^ (Figure 3B). Contractile kinetics were also assessed from high temporal resolution data collected with our force transducer and showed that MM + HPC ECTs had the fastest upstroke velocity (9.93 ± 1.14 × 10^−4^ mN/mm^2^/s), followed by MM ECTs (8.139 ± 0.7045 × 10^−4^ mN/mm^2^/s) (Figure 3C). Relaxation showed similarly fast kinetics in these two treatment groups, where MM ECTs had the shortest time to 50% relaxation (121.4 ± 2.45 ms), followed by MM + HPC ECTs (134.8 ± 10.18 ms) (Figure 3D). Other treatment groups showed lower contractility metrics and are comparable to our prior work [7,41]. These same trends were also observed at 0% strain for active stress generation, upstroke velocity, and time to relaxation (Supplemental Figure S5B), with differences more pronounced at the higher strain percentage.

### 2.4. Mechanical and Structural Changes of ECTs in Hypoxia

**Figure 4:**
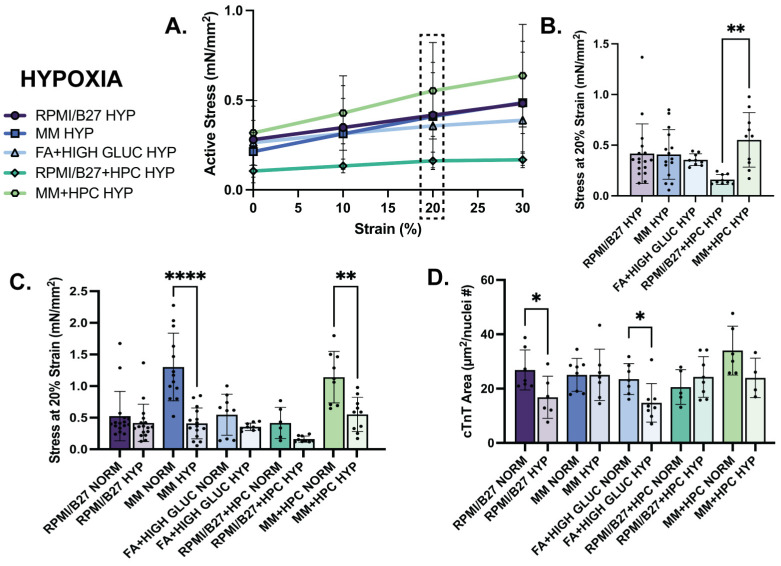
**Hypoxia decreases ECT mechanical function assessed by stress generation across groups, with significant decrease in the MM-treated ECTs despite maintained cTnT area.** (**A**) Active stress generation of hypoxia ECTs from 0 to 30% stretch, measured at increments of 10%. (**B**) Active stress generation of hypoxia ECTs at 20% strain. (**C**) Comparison of active stress generation of normoxia (data reformatted from Figure 3D to be used as comparison) and hypoxia ECTs at 20% strain. (**D**) Comparison of sarcomere area, measured by immunohistological staining of cTnT, normalized by number of nuclei in normoxia and hypoxia ECTs. *n* = 6–17 samples per group for mechanics quantification and *n* = 4–8 samples per group for histology quantification, with significance defined as * *p* < 0.5; ** *p* < 0.01; **** *p* < 0.0001. Dashed box indicates 20% strain, which was the strain used to present the subsequent data. # indicates number.

Improvements in ECT contractility and kinetics observed in MM-treated groups in normoxia were not preserved in hypoxia. After 24 h of hypoxia exposure, the MM stress–strain curve was comparable to the RPMI/B27 and FA + high glucose curves (Figure 4A). Interestingly, though, the MM + HPC group appeared to have the highest active stress for each strain percentage, despite being at ~50% of its normoxic stress generation. At 20% strain, for example, the active stress generation of MM ECTs was not different from the other treatment groups; yet, MM + HPC ECTs displayed the highest active stress generation (0.55 ± 0.09 mN/mm^2^), which was significantly increased compared to RPMI/B27 + HPC ECTs (0.16 ± 0.02 mN/mm^2^) (Figure 4B). This suggests the ability of HPC to partially maintain the enhanced contractile function seen in normoxic ECTs treated with MM. As for contractile kinetics, upstroke velocity was no different among groups at either 20% or 0% strain. However, time to relaxation was decreased in the ECTs treated with MM, as seen in normoxia, suggesting that relaxation was preserved in MM-treated ECTs despite hypoxic stress (Supplement Figure S5C,D). 

When directly comparing treatment groups between normoxia and hypoxia, active stress generation declined in all treatment groups (Figure 4C). Notably, MM-treated ECTs were no longer different from other groups in their active stress generation, showing that the benefit of MM treatment on contractility in normoxia is not maintained in hypoxia (Supplemental Table S1) without further intervention, such as HPC. This response is also shown in the contractile kinetics data (Supplemental Figure S6). There were no statistically significant differences in elastic modulus or cross-sectional area between normoxia and hypoxia within any of the conditions (Supplemental Figure S6), indicating that contractile changes in hypoxia do not result in global morphological or tissue-level passive property changes with 24 h hypoxia exposure. 

While contractile mechanics of ECTs declined to the greatest extent from normoxia to hypoxia in the MM groups, cellular morphologies were impacted most in other treatment groups. Cardiac troponin T (cTnT)-positive myofilament area (normalized by nuclei number) values were not different in the MM groups between normoxia and hypoxia, suggesting that loss of contractile benefit in the MM groups was not due to loss of myofilaments, as may be suggested in other groups (Figure 4D). Nuclear size was quantified in each group as a surrogate measure of nuclear fragmentation which occurs during cell death, and very minor differences from normoxia to hypoxia suggested that there was minimal or no nuclear fragmentation (Supplemental Figure S7).

Electrophysiological parameters and their ability to be maintained in hypoxia across the tested conditions were also assessed through challenge pacing of the ECTs in normoxia and hypoxia. The stress–frequency plots of the ECTs had a negative correlation in both normoxia and hypoxia across all conditions, as expected of hiPSC-CMs [42] (Supplemental Figure S8A,B). Additionally, all ECT conditions across normoxia and hypoxia could follow physiologically relevant pacing of 1–2.5 Hz (Supplemental Figure S8C), with the severity of contractile alternans (periodic alternation between full-force and partial-force contraction, quantified as the difference in force normalized to the full-force) being less than 8% up to 2.5 Hz (Supplemental Figure S8D–G). 

### 2.5. Metabolic Functional Changes of ECTs Occurring during Hypoxia

**Figure 5:**
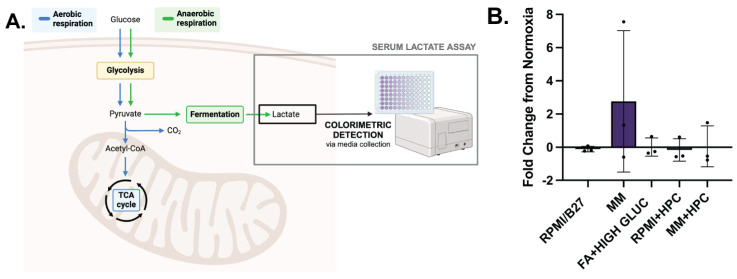
**MM-treated ECTs undergo a metabolic functional shift during hypoxia which can be impacted by HPC.** (**A**) Colorimetric detection assay to identify L-lactate concentration, an indicator of anaerobic glucose metabolism and cell physiological stress. (**B**) L-lactate fold change from normoxia conditions, with normalization to the number of tissues and hours of culture. *n* = 3 per group. Created with BioRender.com.

To evaluate dynamic metabolic changes occurring during hypoxia, a serum lactate assay was used to detect concentration of L-lactate, a product of anerobic glucose metabolism, in the media of different groups in normoxia and hypoxia to identify any metabolic shifts. L-lactate is produced during anaerobic glucose metabolism (Figure 5A), which does not require oxygen, and is the primary metabolic pathway used by immature CMs. Metabolically mature CMs, on the other hand, primarily use oxidative metabolic pathways, which have increased ATP production. Fold change in L-lactate produced during hypoxia was quantified (Figure 5B), with MM ECTs having the greatest increase in L-lactate production from normoxia to hypoxia (2.77 ± 2.46; n.s.). This likely indicates a shift from fatty acid oxidation in normoxia (no L-lactate production) to glycolysis in hypoxia (increased L-lactate production). Interestingly, MM + HPC did not have an increase in L-lactate production during hypoxia, suggesting the ability of HPC to shift CM metabolism toward glycolysis prior to prolonged hypoxia. 

## 3. Discussion

At its core, MI is a disease characterized by oxygen imbalance. During acute MI, the oxygen supply of the damaged vasculature cannot meet the high demand of the functional cardiomyocytes, resulting in a highly ischemic and hostile environment [8,9]. In developing cell-based therapies for heart regeneration post-MI, in which SC-CMs are implanted into this environment, consideration of ischemia, as well as how it interacts with implanted CMs, is imperative to push the clinical translation of this technology successfully forward. In this study, we demonstrate the use of in vitro culture techniques to influence the hypoxia resistance of hiPSC-CMs. We identify metabolic conditioning and HPC as two treatments that can significantly influence the hypoxic response of hiPSC-CMs in 2D cultures and 3D ECTs. Metabolic conditioning promoting oxidative phosphorylation is shown to significantly increase active stress generative in ECTs compared to other treatment conditions; yet, this functional enhancement is lost in hypoxia. Interestingly, however, HPC can be used prior to hypoxia to maintain some of the enhanced contractile function of metabolically matured hiPSC-CMs. To our knowledge, this is the first systematic testing of in vitro culture conditions to directly compare their impact specifically on hypoxic response and resistance of hiPSC-CMs. These results provide insight to better understand the interplay between hiPSC-CMs and hypoxia, engineering techniques to culture hiPSC-CMs with greater ischemia resistance, and the development of more robust cell-based cardiac therapies. 

The ability to differentiate SC into CMs provides a virtually limitless human cell source, which is ideal for cardiac regenerative therapies to replace the massive quantity of CMs lost during MI. An important aspect of this cell population, however, is their immaturity compared to adult CMs [12,43]. Work performed by Uosaki et al. (2015) mapped the transcriptional landscape of SC-CMs during differentiation (up to day 10) and subsequent in vitro culture (up to day 30) against that of the developing heart, finding that SC-CMs arrested at a late embryonic stage of development [12]. However, it has been well established in the field of cardiac tissue engineering that the in vitro culture conditions of SC-CMs are able to significantly push forward the maturation of SC-CMs in terms of structure [44,45,46,47], metabolism [13,27,28,29,30], transcriptomics [46,48], and overall function [29,46,47]. In this work, we demonstrate the ability of metabolic conditioning to improve the contractile maturation of our ECTs, as quantified by increased active stress generation and improved contractile kinetics, which is well corroborated by others in the field [13,27,28,29,30]. 

Maturation of SC-CMs is imperative in applications of SC-CMs within disease modeling and pharmacological studies. For example, differences in expression levels, isoforms, localization, and function of SC-CM ion channels compared to adult CMs [12,49,50,51] limit their ability to detect drug or disease-induced arrhythmias that impact the activity of mature ion channels [52,53]. The extent of SC-CMs required for cell regeneration applications, however, is less obvious. Pivotal studies by Romagnuolo et al. [54], Chong et al. [55], and Liu et al. [56] have reported the occurrence of engraftment arrythmias (EAs) upon injection of SC-CMs into pig and non-human primate hearts. These EAs are characterized as life-threatening ventricular tachyarrhythmias which originate from SC-CMs engraftment sites. It is hypothesized that the electrophysiological immaturity of these SC-CMs is in part to blame, as ventricular SC-CMs have substantial automaticity, depolarized mean diastolic potential, and shorter action potentials, which likely contribute to such arrythmia generation [43]. Although these studies conclude that a more mature phenotype for SC-CMs is needed in regenerative therapies, in practice it is more nuanced. In a study by Peters et al., metabolically matured SC-CMs displayed a significant increase in cell death as well as mitochondrial dysfunction compared to non-matured SC-CM controls [17]. However, Dhahri et al. et al. matured their SC-CMs through culture on a soft PDMS substrate, which resulted in enhanced structure, host–graft electromechanical integration, and contractile contribution, as well as less proarrhythmic behavior upon injection in a guinea pig model of MI [15]. Further, it has been shown that immature SC-CMs are able to readily engraft in the heart post-MI [57], and regardless of maturation state before implantation, SC-CMs undergo impressive maturation in terms of sarcomere development, organization, and isoform, demonstrated through histological staining [41,55,56,58]; electrical quiescence and host coupling, as shown by EAs falling to zero after 4 weeks in vivo [54,56,58]; and transcriptional shifts toward a more matured phenotype, detected in RNA sequencing [41]. Taken as a whole, this work points to the following notions: (1) SC-CM maturation occurs in a multi-factorial, networked manner, rather than a linear trajectory; (2) different in vitro techniques can tune CM maturation though different nodes within this network; and (3) there is an increasing need to clearly define the most appropriate or “ideal” maturation state of SC-CMs specifically for regenerative therapies. Our current study represents a first step in defining an “ideal” maturation by assessing the impact of different in vitro culture conditions on ECT formation, function, and response to hypoxia. 

As described above, metabolic conditioning has emerged as a potent pathway to mature SC-CMs, yet seems to impede the engraftment success of SC-CMs. Several published papers in the field confirm that there is a critical link between metabolism and CM maturation [13,27,28,29,30]. During heart development, there is a major metabolic shift from glycolysis in fetal CMs to oxidization phosphorylation after birth to accommodate the tremendous energy demands of the heart. Interestingly, this shift is accompanied by significant cellular changes including increased oxygen demand; enhanced ATP production; accumulation of reactive species (ROS) production that is followed by DNA damage and cell cycle exit; hypertrophy of CMs with alignment of sarcomeres; and mitochondrial fusion and biogenesis [59,60]. Traditional (and field-standard) culture of SC-CMs utilizes RPMI/B27, which features glucose (~11.1 mM) rather than fatty acids (<10 mM total [27,61]) as the main energy source. Yet, shifting the metabolism of SC-CMs has been achieved in vitro by supplementing the medium with different metabolic substrates [13,27,28]. On one hand, this maturation is attractive as it is easy to implement in vitro, potent, and able to propel the mechanical and electrical properties of the cultured SC-CMs toward that of native myocardium. On the other hand, such maturation also means SC-CMs have a higher oxygen demand, which may not be met in the post-MI hypoxic environment. In our work, we report that although functional benefit from metabolic conditioning of ECTs is significant in normoxia, it is lost with exposure to hypoxia. Having both metabolic substrates to promote oxidative phosphorylation (i.e., fatty acids) and glycolysis (i.e., high glucose) in anticipation of the metabolic shifts that may occur during hypoxia did little to either promote contractile function of ECTs or curb its decline in hypoxia compared to controls. Our experiments maintained the metabolic conditioning media during hypoxia, so future work could evaluate the impact of early metabolic conditioning as well as limited substrate availability during hypoxia on hiPSC-CM function. 

A critically important finding of our study is that HPC partially rescues function of metabolically matured ECTs to provide greater contractility than control (non-metabolically matured) tissues in hypoxia. Preclinical studies throughout the field utilizing HPC (also known as intermittent hypoxia [36]) have demonstrated its ability to reduce myocardial damage [62,63,64,65,66], decrease ventricular arrythmias [62,63,64,67] and improve outcomes [66,68,69,70] in animal models of MI. It is hypothesized that hypoxia conditioning enables CMs to activate a variety of cardioprotective mechanisms which allow them to survive in prolonged ischemia. Some of these mechanisms include: increased glycogen storage for prolonged ATP production in ischemia [71]; preservation of calcium handling due to upregulation of calcium-binding proteins [72,73]; increased ion channel density to resist their inactivation during prolonged ischemia [74]; stabilization of the mitochondria through inhibition of mitochondrial permeability transition pores (MPTP) opening [75]; and activation of two potent transcriptional factors, hypoxia-inducible factor 1 (HIF-1) and nuclear factor erythroid-2 related factor 2 (Nrf2), which trigger the expression of a variety of cardioprotective proteins [36,37,38,39]. Despite its ability to protect CMs, HPC has not been adopted clinically due to its perceived risk to patients and the uncertainties of when to administer such a treatment [36]. Yet, for in vitro applications, HPC is a very promising technique, as it can be easily adopted into current workflows and is able to trigger a complex cascade of protective signaling. In this way, HPC has been widely applied in the development of extracellular vesicles (EVs) for cardiac therapies. Several groups have shown that EVs collected from cells cultured in hypoxic conditions demonstrate enhanced ability to preserve CM function after injury compared to normoxic EVs [76,77,78,79]. In this study, we are the first to use HPC in a tissue engineering application. We applied a mild HPC conditioning protocol, consisting of 30 min of hypoxia, which we ensured did not induce cell death (Figure 1E), 24 h prior to prolonged hypoxia. While this HPC protocol was able to slightly improve ECT hypoxia resistance, others in the field have shown HPC timing and duration has a significant effect on CM protection [36,80,81,82]. This suggests that further optimization of HPC severity (i.e., oxygen % timing, and duration) could yield an even higher degree of hypoxia resistance within our ECTs. 

To date, little attention has been given to hypoxia resistance of SC-CMs for remuscularizing the heart, while many approaches to vascularization in the post-MI environment are being pursued. We agree that increased angiogenesis would reduce the duration and/or severity of hypoxia experienced by implanted SC-CMs and must also be pursued. Approaches to revascularization post-MI are diverse, including the delivery of proangiogenic factors (i.e., growth factors, EVs, miRNA) alone or in conjunction with cell-based approaches (i.e., endothelial implantation, patterned vasculature, cell-based paracrine signaling) [83,84,85]. In the area of revascularization coupled to cardiac regenerative therapies delivering SC-CMs, our lab has shown that two different approaches successfully increase the perfused vascular network within the ECT. First, using biomaterials to locally release angiogenic factors from the implant increases vessel density, branching, and length with reduced tortuosity [86]. Second, pre-vascularized patterned vessel-like networks formed within hiPSC-CM ECTs increase the density of implant vascularization [41], which is well corroborated by others using bulk seeding of endothelial cells within ECTs [87,88,89]. Further, our approach improved hiPSC-CM engraftment density, and for the first time in natural materials, our patterned vessels were used by penetrating vascular cells and perfused at their full diameter (150–250 µm) in one of three hearts at two weeks. Even with these approaches to increase rates of angiogenesis, however, implanted SC-CMs will experience some level of hypoxia, since it takes time for developing vessels to vascularize the implanted ECT, even if patterned vessels enable a more rapid anastomosis for convective perfusion of the ECT. Importantly, the results of our current study demonstrate that while SC-CMs intrinsically resist hypoxia damage, complete cell death was 1.20 ± 0.27% after 12 h of hypoxia exposure at 1% O_2_ (Supplemental Figure S3D–E), consistent with results from others [17,40], and that metabolic function decreased by more than 30% after just 2 h of hypoxia (Figure 1A, Control). Ultimately, SC-CM efficacy as a therapy may be severely limited if the functional benefit of implanted SC-CMs is compromised by prolonged hypoxia. Thus, a promising approach, especially as ECTs become larger and thicker to accommodate a higher therapeutic load of SC-CMs [5,7], is a combinatory approach leveraging both hypoxia resistance and promotion of vascularization. In conclusion, this study demonstrates that hypoxic preconditioning of SC-CMs in vitro engineers these cells to accommodate some level of hypoxia while maintaining function, which may be critically important for maximizing long-term therapeutic efficacy with co-revascularization approaches to ensure that the hypoxic duration is limited.

## 4. Materials and Methods

### 4.1. Human Induced Pluripotent Stem Cell (hiPSC) Maintenance

The WTC-11 GCaMP hiPSC line (Bruce Conklin, Gladstone Institute, UCSF) was used for all cardiac differentiations. For maintenance of hiPSCs, cells were seeded into vitronectin-coated (5 µg/mL) 10 cm^2^ tissue culture-treated plates (Thermo Fisher, Waltham, MA, USA) and maintained in Essential 8 (E8) media (Gibco) in an incubator (37 °C, 5% CO_2_). At 80% confluency (4–5 days), cells were passaged using Versene (0.5 M EDTA, 1.1 mM D-glucose; MilliporeSigma, Cleveland, OH, USA) in Dulbecco’s phosphate-buffered saline (DPBS; Gibco) without calcium and magnesium.

### 4.2. HiPSC-CM Differentiation, Freezing/Thawing, and Expansion

**Figure 6:**
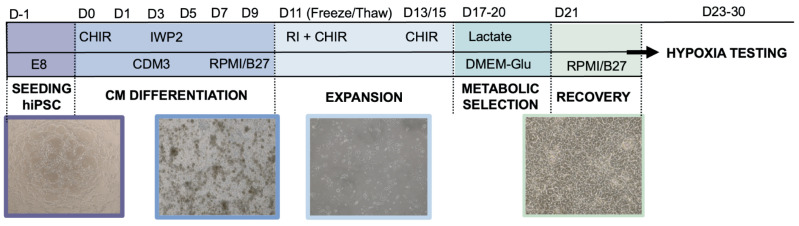
**Protocol for differentiation of human induced pluripotent stem cells (hiPSC) into cardiomyocytes (CMs) and subsequent culture.**

Cardiomyocytes were terminally differentiated from hiPSCs (hiPSC-CMs) and cultured on standard, Geltrex-coated (0.1 mg/mL, Gibco) tissue culture plastic according to previously published protocols [18,90,91]. Briefly, cardiomyocytes were differentiated from hiPSCs through biphasic control of the Wnt signaling pathway; expanded for six days using a low concentration of Wnt activator CHIR 99021 (Chiron; Tocris, Bristol, UK); and lactate-purified using a glucose-depleted culture medium (DMEM–glucose, Gibco) supplemented with 2 mM sodium lactate (MilliporeSigma, Cleveland, OH, USA). HiPSC-CMs were then maintained in RPMI/B27 with media being replenished every other day (Figure 6). For treatment conditions requiring replating onto a new substrate, hiPSC-CMs were harvested using TrypLE Select Enzyme (10X, Gibco) and seeded with 10 µM Y-27632 (Rock Inhibitor, RI; Tocris, Bristol, UK). HiPSC-CMs were frozen in CryoStor^®^CS10 (Stem Cell, Vancouver, BC, Canada) on D11 to decouple differentiation and expansion for downstream application processes. This protocol yielded cardiomyocyte purity > 53.44% quantified by flow cytometry analysis of cardiac troponin T (cTnT) expression, with a range of 53.44–88.99% used in 2D experiments, and a range of 61.8–90.02% used in 3D experiments. Fold change from internal controls was used to account for variability in purity.

### 4.3. In Vitro Treatment for 2D hiPSC-CM Hypoxia Experiments

HiPSC-CM treatment conditions are summarized in Table 1. All experiments were repeated with at least three biological replicates.

### 4.4. Damage Induction

HiPSC-CMs were exposed to hypoxic conditions (1% O_2_, 5% CO_2_, EVOS Cell Imaging System Hypoxia Chamber) for 2 h (2D MTT experiments), 3–12 h (2D LIVE/DEAD experiments), or 24 h (3D experiments).

### 4.5. MTT Assay

A MTT assay was performed to quantify cell activity based on metabolic dysfunction during hypoxia. The MTT stain (MilliporeSigma, In Vitro Toxicology Assay Kit, MTT Based) was applied according to the manufacturer protocol. An incubation time of 2 h was used before the solubilization solution was applied. The absorbance of each well (*n* = 5 wells per condition for 96-well plate, n = 3 for 12-well plate, n = 3–4 wells for 6-well plate) was measured using a plate reader (BioTek Cytation 5 Cell Imaging Multimode Reader, Agiliant, Santa Clara, CA, USA).

### 4.6. LIVE/DEAD Assay and ImageJ Analysis

A LIVE/DEAD assay was performed to quantify cell death during hypoxia. The LIVE/DEAD stain (ThermoFisher Scientific, ReadyProbes Cell Viability Imaging Kit (Blue/Red)) was applied prior to hypoxia according to the manufacturer protocol. The NucBlue live reagent stains the nuclei of all cells and can be visualized using a standard DAPI feature, while the propidium iodide dead reagent stains cells with compromised plasma membrane integrity and can be visualized using a standard RFP filter. Images were captured prior to hypoxia and every hour during hypoxia in one location per well. ImageJ (Version 1.0) was used to binarize images and count the total number of cells and the number of dead cells.

### 4.7. Cardiac Fibroblast (hCF) Maintenance and Freezing/Thawing

Human primary ventricular cardiac fibroblasts (hCFs; Lonza, Basel, Switzerland) were seeded into 15 cm^2^ plates and maintained in media containing DMEM/F12 (Gibco), 10% FBS, 4 ng/mL bFGF (Stemgent, Beltsville, MD, USA), and 100 µg/mL penicillin-streptomycin (penstrep; MilliporeSigma). At confluency, hCFs were passaged using 0.05% trypsin (Gibco) in Versene and frozen back in hCF media with 10% DMSO.

### 4.8. Polydimethylsiloxane (PDMS) Mold Fabrication

PDMS molds were fabricated for casting and culture of ECTs. A previously published replica-molding protocol was used [93]. Briefly, vector-based designs were created in Adobe Illustrator and a laser cutter (Universal Laser Systems 6.75 Laser Cutter (ULS), Brown Design Workshop (Brown University, Providence, RI, USA)) was used to etch the negative template into ¼ inch acrylic sheet. PDMS (Sylgard 184) was then cast onto the acrylic negative template. A vacuum chamber was used to remove any bubbles, and the PDMS was cast at 60 °C overnight. Once the PDMS was set, the molds were removed from the template and sterilized using an autoclave that reached 121 °C for 30 min. For this study, linear molds (3 × 9 mm, 35 µL tissue casting volume) were used. Finally, 5% Pluronic^®^ F-127 (MilliporeSigma) was used to coat PDMS molds for one hour prior to tissue casting in order to prevent adhesion to the mold and non-homogeneous tissue formation.

### 4.9. ECT Fabrication

HiPSC-CMs were harvested using TrypLE Select Enzyme (10X) and combined with 5% hCFs from thaw. The cell solution was diluted in RPMI/B27 media to a concentration of 30 million cells per mL. Then, a collagen hydrogel (2 mg/mL) was prepared using a commercial stock of rat tail type I collagen (3.9–4.1 mg/mL; Advanced BioMatrix, Carlsbad, CA, USA), 10X RPMI 1640 (1:10; Gibco), HEPES (1:100; MilliporeSigma, Cleveland, OH, USA), mH_2_O, and 1 M sodium hydroxide to a pH between 7 and 7.5. The cell solution and collagen solution were then combined in a 1:1 ratio and immediately cast into PDMS molds. After a 30–45 min period for the gel to set, cell culture media was added. Several media formulations were used (Table 2), with media being replaced on alternate days.

### 4.10. In Vitro Treatment of ECTs

ECT treatment conditions are summarized in Table 2. All experiments were repeated with at least three biological replicates.

### 4.11. ECT Survival and Compaction Analysis during Culture to Assess Syncytium Formation

Brightfield images (Olympus SZ40, Shinjuku, Tokyo, Japan) were captured every day for the seven days of ECT culture prior to hypoxia. Survival curves were generated by quantifying ECT breakage, with intact ECTs being defined as having no breakage either internally or at the PDMS posts. To evaluate ECT compaction, ImageJ was used to measure the tissue area on each day, with tissue area normalized to day 0 area.

### 4.12. Immunohistochemical Staining and Imaging of ECTs to Assess Changes in Structure

Histological staining was performed as previously described [7,41]. ECTs were fixed in 4% paraformaldehyde (MilliporeSigma) and washed with DPBS. For short-term storage, samples were kept at 4 °C. For staining, ECT samples were frozen and embedded in optical cutting temperature medium, followed by sectioning at 5 µm. ECTs were sectioned lengthwise through the full thickness of the tissue. Multiple sections throughout the ECT thickness (≥3) were used for staining.

For immunohistochemical staining, sectioned samples were blocked with 1% normal goat serum (NGS; MilliporeSigma) in DPBS for 1 h followed by incubation with primary antibody cardiac troponin T (cTnT), myosin regulatory light chain 2, ventricular isoform (MLC2v), and/or myosin regulatory light chain 2 and atrial isoform (MLC2a) overnight at 4 °C. The following day, sections were incubated with secondary antibodies and nuclear counterstains for 1 h at room temperature. Coverslips were mounted using Prolong AntiFade Glass Mountant (Invitrogen, Waltham, MA, USA). Once set, sections were imaged using an Olympus FV3000 confocal microscope (Shinjuku, Tokyo, Japan) and quantified in ImageJ.

### 4.13. Mechanical Testing of ECTs to Analyze Functional Changes in Stress Generation and Kinetics

A custom micromechanical tensile apparatus (Aurora Scientific, Aurora, Canada) was used to analyze the passive and active mechanics of ECTs by quantifying Young’s modulus and contractile stress generation (Supplemental Figure S1A). After 7 days in 3D culture, tissues were analyzed (normoxia controls) or underwent 24 h of damage induction (hypoxia). To analyze their mechanics, ECTs were mounted onto the tensile apparatus in a 37 °C bath of Tyrode’s solution (Supplemental Figure S1B). Tissues were stretched to 130% (10% beyond the physiological relevant strain of 20% to assess potential application in pathological stretch) of their initial length in 10% increments and maintained at each strain for 120 s to allow for stress relaxation (Supplemental Figure S1C). An electrical stimulus of 1 Hz was administered to determine active stress contraction at every tested strain. At the final stage (30% strain), the force-frequency response of the tissue was determined by increasing the electrical stimulus from 1 Hz to 4 Hz in 0.5 Hz intervals. A MATLAB (Mathworks, Natick, MA, USA) script was utilized to analyze active contractile amplitude (force, mN; active stress generation, mN/mm^2^) and kinetics (upstroke velocity, mN/mm^2^/s; time to 50% relaxation and 90% relaxation, ms). Contractile alternans (change observed in contractile amplitude) were also quantified as difference between amplitude in adjacent contractions normalized by the greater contraction. The force–frequency response was recorded, with the maximum capture rate (MCR) defined as the maximum frequency at which the ECT could follow the target pacing.

### 4.14. Serum Lactate Assay for ECTs to Assess Functional Changes in Metabolism during Hypoxia

L-lactate production of ECTs (an indicator of anaerobic metabolism and physiological stress) was assessed in normoxic and hypoxic culture conditions. Cell culture media was collected after 40–48 h of normoxic culture, and after 24 h of hypoxic culture. For culture media already containing L-lactate, samples were diluted 1:100 in DMEM media. Duplicates of each sample, as well as fresh media controls, were quantified in terms of lactate concentration using L-lactate Colorimetric Assay Kit (RayBiotech, Peachtree Corners, GA, USA) according to the manufacturer protocol. A plate reader was used to measure the absorbance at 510 nm, and a standard curve was used to convert absorbance to concentration. Dilution was then accounted for, and concentrations were scaled by the number of hours in culture and the number of ECTs per well.

### 4.15. Calculation of Fold Change

Fold change was calculated to quantify changes from normoxia (before damage induction; initial value) to hypoxia (after damage induction; final value). Because the tested metrics almost exclusively decrease from normoxia to hypoxia, all fold changes for MTT, LIVE/DEAD, and mechanics experiments were multiplied by negative one in order to obtain positive values. Thus, fold change was calculated using the following equation:

$$Fold\;change=-\frac{final-initial}{initial}=-\frac{hypoxia-normoxia}{normoxia}$$

Positive values of fold change, as calculated above, indicate decreases in cell viability or tissue properties from normoxia to hypoxia (expected), with larger magnitude, positive values indicating larger decreases. Negative values indicate improvement from normoxia to hypoxia, with larger magnitude, negative values indicating larger increases (a few select cases).

For serum lactate experiments, fold change was not multiplied by negative one, and was calculated using the following equation:

$$Fold\;change=\frac{final-initial}{initial}=\frac{hypoxia-normoxia}{normoxia}$$

Positive values of fold change indicate increases in L-lactate production, while negative values indicate decreases in L-lactate production.

### 4.16. Statistical Analysis

Student *t*-tests as well as one-way or two-way repeated-measures analysis of variance (ANOVA) were used. When necessary, the Tukey–Kramer method of post hoc analysis was performed, with *p*-values < 0.05 considered statistically significant. All analysis was performed in Prism 8 (GraphPad, Boston, MA, USA) and reported with the standard error of the mean.

## 5. Conclusions

As the field moves to translate ECTs from bench to bedside, hiPSC-CM response to hypoxia becomes crucial for successful implant engraftment to the infarcted myocardium. In this study, we found that metabolic conditioning and hypoxic preconditioning (HPC) had significant influence on hiPSC-CM function in normoxic and hypoxic conditions, which guides our strategy for preparing hiPSC-CMs for transplantation. Our data supports the use of a maturation medium (MM) with palmitate and physiological calcium levels to maximize metabolic and contractile function in normoxia. However, these moderately matured hiPSC-CMs are more vulnerable to hypoxia. To benefit from in vitro maturation and protect against hypoxic stress, exposure of MM-treated hiPSC-CMs to HPC increases resilience and enables greater preservation of metabolic and contractile function in hypoxia. Ultimately, a balance between hiPSC-CM maturation/contractility and ischemia resistance is crucial to maximizing the therapeutic benefit of ECTs, and this study identifies an engineered strategy to achieve this outcome through combined MM and HPC treatments.

## Figures and Tables

**Table 1:** Summary of in vitro hiPSC-CM treatment conditions.

Metabolic conditioning	HiPSC-CMs were seeded into Geltrex-coated (0.1 mg/mL) 96-well plates (10,000–12,000 cells/well). After an overnight recovery period, hiPSC-CMs were switched to five different cell culture media for one week prior to hypoxia (*n* = 5 wells per condition). The media compositions were as follows: (1) RPMI/B27 (control; high glucose, 11.1 mM), (2) high glucose/high FA (RPMI/B27 + 0.5% *w*/*v* Albumax (Thermo Fisher Scientific, Waltham, MA, USA) + 2 mM L-Carnitine (Sigma Aldrich, St. Louis, MO, USA)), (3) low glucose/high FA (RPMI/B27–glucose + 3 mM glucose (Sigma Aldrich) + 0.5% *w*/*v* Albumax + 2 mM L-Carnitine), (4) FA only (RPMI/B27–glucose + 0.5% *w*/*v* Albumax + 2 mM L-Carnitine), (5) Maturation media (MM) [13] (DMEM without glucose (Thermo Fisher Scientific) supplemented with 3 mM glucose (Sigma Aldrich), 10 mM L-lactate (Sigma Aldrich), 5 μg/mL Vitamin B12 (Sigma Aldrich), 0.82 μM Biotin (Sigma Aldrich), 5 mM Creatine monohydrate (Sigma Aldrich), 2 mM Taurine (Sigma Aldrich), 2 mM L-carnitine (Sigma Aldrich), 0.5 mM Ascorbic acid (Sigma Aldrich), 1× NEAA (Thermo Fisher Scientific), 0.5% (*w*/*v*) Albumax (Thermo Fisher Scientific), 1× B27 and 1% KOSR (Thermo Fisher Scientific)).
Wnt signaling	HiPSC-CMs were seeded into Geltrex-coated (0.1 mg/mL) 96-well plates (10,000–12,000 cells/well). After 24 h, hiPSC-CMs were treated with 4 μM dimethyl sulfoxide (DMSO, control; Fisher Scientific, Waltham, MA, USA), 2 μM Chiron (Wnt activation), or 4 μM C59 (Wnt inhibition, Tocris) overnight (*n* = 8 wells per condition for LIVE/DEAD; *n* = 5 wells per condition for MTT).
Mechanics (PDMS)	A thin layer (<0.5 mm) of polydimethylsiloxane (PDMS) (Sylgard 184 and Sylgard 527 in a 2:1 ratio, Dow, Midland, MI, USA) was glued into the bottom of a 12-well tissue culture plate using Dow sealant mixed with ethanol. The PDMS was treated with sterile-filtered polydopamine (PDA, MilliporeSigma) solution (0.01% (*w/v*) in 1 M Tris-HCl buffer (pH 8.5) overnight at 4 °C [92]. The PDA was then aspirated, the PDMS was washed with sterile-filtered mH_2_O twice, and finally coated with Geltrex (0.1 mg/mL) overnight at 4 °C. HiPSC-CMs were seeded onto PDMS substrate or standard Geltrex-coated tissue culture plastic (110,000 cells/well) and maintained for one week prior to hypoxia (*n* = 3 wells per condition).
Alignment (PCL)	Nano-aligned PCL scaffolds (Nanofiber Solutions, Dublin, OH, USA) were glued into the bottom of a 12-well tissue culture plate using Dow sealant mixed with ethanol and then coated with Geltrex (0.1 mg/mL) overnight at 4 °C. HiPSC-CMs were seeded onto PCL substrate or standard Geltrex-coated (0.1 mg/mL) tissue culture plastic (110,000 cells/well) and maintained for one week prior to hypoxia (*n* = 3 wells per condition).
Electrical pacing (EP)	HiPSC-CMs were seeded into a Geltrex-coated (0.1 mg/mL) 6-well cell culture plate (300,000 cells/well). After 24 h, hiPSC-CMs were subjected to electrical pacing or no electrical pacing (*n* = 3–4 wells per condition). Electrical paced hiPSC-CMs were stimulated with a 4 ms biphasic field pulse stimulus at 1 Hz and 4 V/cm using a 6-well electrode insert (C-Dish, IonOptix, Westwood, MA, USA) connected to the IonOptix culture pacing system (C-Pace EP, IonOptix, Westwood, MA, USA).
Heat shock (HS)	HiPSC-CMs were seeded into Geltrex-coated (0.1 mg/mL) 96-well plates (10,000–12,000 cells/well). After 24 h, hiPSC-CMs were subjected to one hour of heat shock at 42 °C. Four groups (n = 5 wells per condition) were tested in hypoxia: (1) Control (37 °C, normoxia), (2) heat shock only (42 °C, normoxia), (3) hypoxia only (37 °C, hypoxia), and (4) heat shock + hypoxia (42 °C, hypoxia).
Hypoxia preconditioning (HPC)	HiPSC-CMs were seeded into Geltrex-coated (0.1 mg/mL) 96-well plates (10,000–12,000 cells/well). After an overnight recovery period, hiPSC-CMs were subjected to 30 min of hypoxia (1% O_2_, 5% CO_2_) 24 h prior to damage induction. Four groups (*n* = 5 wells per condition) were tested in hypoxia: (1) Control (no pre-exposure, no hypoxia), (2) pre-exposure only, (3) hypoxia only, and (4) pre-exposure and hypoxia.
Apoptosis inhibition (cyclosporine)	HiPSC-CMs were seeded into Geltrex-coated (0.1 mg/mL) 96-well plates (10,000–12,000 cells/well). After 24 h, hiPSC-CMs were treated with 0.1 μM Cyclosporine A overnight prior to hypoxia or left as untreated controls (*n* = 5 wells per condition) [20].
Mitochondrial stabilization (Mito-TEMPO)	HiPSC-CMs were seeded into Geltrex-coated (0.1 mg/mL) 96-well plates (10,000–12,000 cells/well). After 24 h, hiPSC-CMs were treated with 25 μM Mito-TEMPO (MilliporeSigma) overnight prior to hypoxia or left as untreated controls (*n* = 5 wells per condition) [19].

*n* reported per biological replicate per condition.

**Table 2:** Summary of in vitro ECT treatment conditions.

RPMI/B27 (high glucose media)	ECTs were cultured for seven days, with RPMI/B27 with media being replaced on alternate days.
Maturation medium (MM)	ECTs were cultured for seven days, with MM (Table 1) [13] being replaced on alternate days.
FA + high glucose media	ECTs were cultured for seven days, with FA + high glucose media (RPMI/B27 + 0.5% *w*/*v* Albumax + 2 mM L-Carnitine) being replaced on alternate days.
RPMI/B27 + HPC	ECTs were cultured for seven days, with RPMI/B27 (Thermo Fisher Scientific) media being replaced on alternate days. On day 6, ECTS were exposed to 30 min of hypoxia (1% O_2_, 5% CO_2_) for 30 min approximately 24 h before hypoxia damage induction, and then returned to the incubator.
MM + HPC	ECTs were cultured for seven days, with MM (Table 1) [13] being replaced on alternate days. On day 6, ECTS were exposed to 30 min of hypoxia (1% O_2_, 5% CO_2_) for 30 min approximately 24 h before hypoxia damage induction, and then returned to the incubator (normoxia).

## Data Availability

Data may be made available upon request.

## Supplementary Materials

The following supporting information can be downloaded at: https://www.mdpi.com/article/10.3390/ijms25179627/s1.
