# Supplementary material for: Advancing Human iPSC-Derived Cardiomyocyte Hypoxia Resistance for Cardiac Regenerative Therapies through a Systematic Assessment of In Vitro Conditioning
Source: Int J Mol Sci. 2024 Sep 5;25(17):9627. doi: 10.3390/ijms25179627 (PMC11395605; doi:10.3390/ijms25179627)
Supplement: Supplementary file 1 [file ijms-25-09627-s001.zip › ijms-3171333-supplementary.pdf]

**Supplemental Figures**

**Supplemental Figure S1.** Mechanical testing of ECT setup and protocol.
**Supplemental Figure S2.** MTT Assay Cell validation and setup.
**Supplemental Figure S3.** LIVE/DEAD assay for 3-12 hours of hypoxia
**Supplemental Figure S4.** Immunohistochemical staining of ECTs.
**Supplemental Figure S5.** Active stress generation and kinetics of normoxic and hypoxic ECTs.
**Supplemental Figure S6.** Comparing ECT active stress generation and kinetics after hypoxia treatment.
**Supplemental Figure S7.** Quantification of nuclei size in normoxic and hypoxic ECTs.
**Supplemental Figure S8.** Stress-Frequency response of ECTs in normoxia and hypoxia.

**Supplemental Table S1.** Fold change in active stress generation (mN/mm2), upstroke velocity (Vup, mN/mm2/s), and time to 50% relaxation (T50, relaxation) from normoxia to hypoxia.

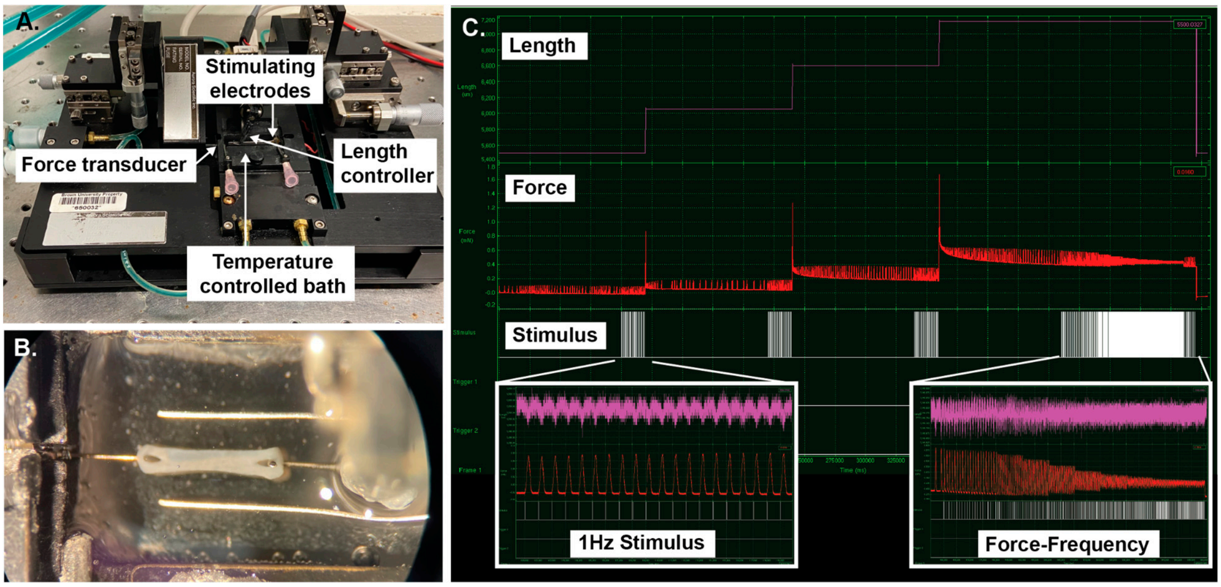


**Supplemental Figure S1. Mechanical testing of ECT setup and protocol. (A)** Micromechanical tensile apparatus (Aurora Scientific, Aurora, Canada) consisting of 5mN force transducer, length controller, 37°C temperature-controlled bath and stimulating electrodes; **(B)** ECT mounted on the force transducer (left) and length controller (right) for mechanical testing; **(C)** Mechanical protocol of length steps 0%, 10%, 20% and 30% with 1Hz stimulation delivered at the end of each length step. At 30% strain, force frequency response is measured by increasing frequency of stimulation by 0.5Hz.

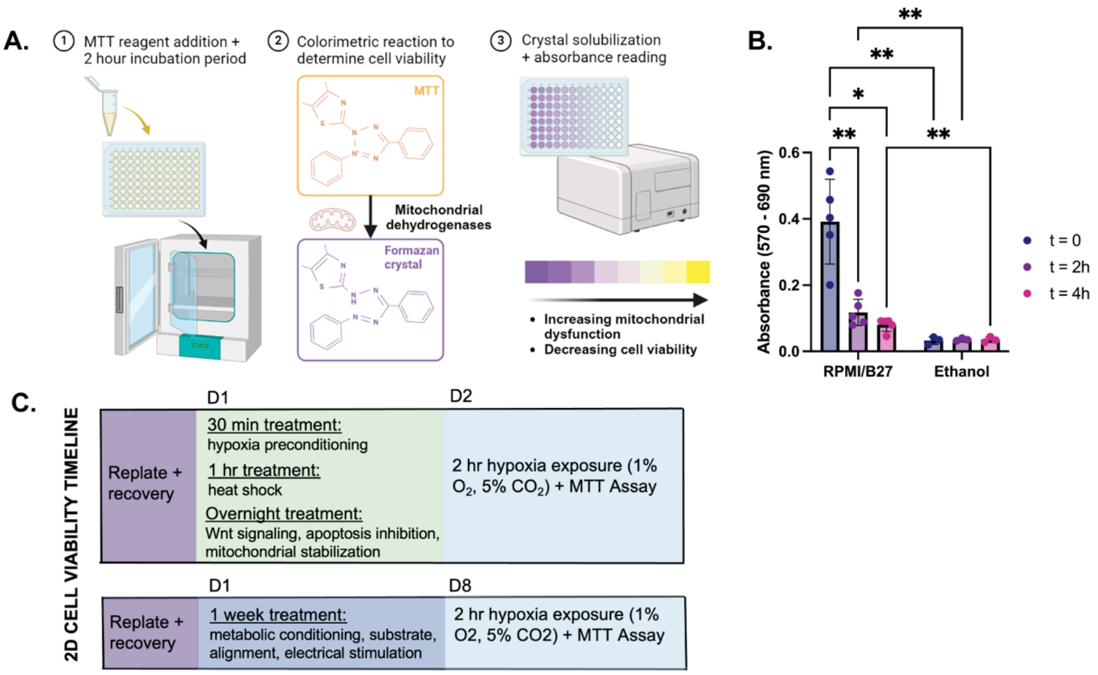


**Supplemental Figure S2. MTT Assay Cell validation and setup. (A)** Schematic of MTT Assay mechanism and quantification; **(B)** Absorbance of hiPSC-CMs treated with RPMI/B27 or ethanol (positive control for cell death) at t = 0, 2, 4h (*n* = 3-5 per group); **(C)** Timeline of 2D cell culture, treatment, and damage induction. (*n* = 9-15 per group). * $p < 0.05$; ** $p < 0.01$. Made with BioRender®

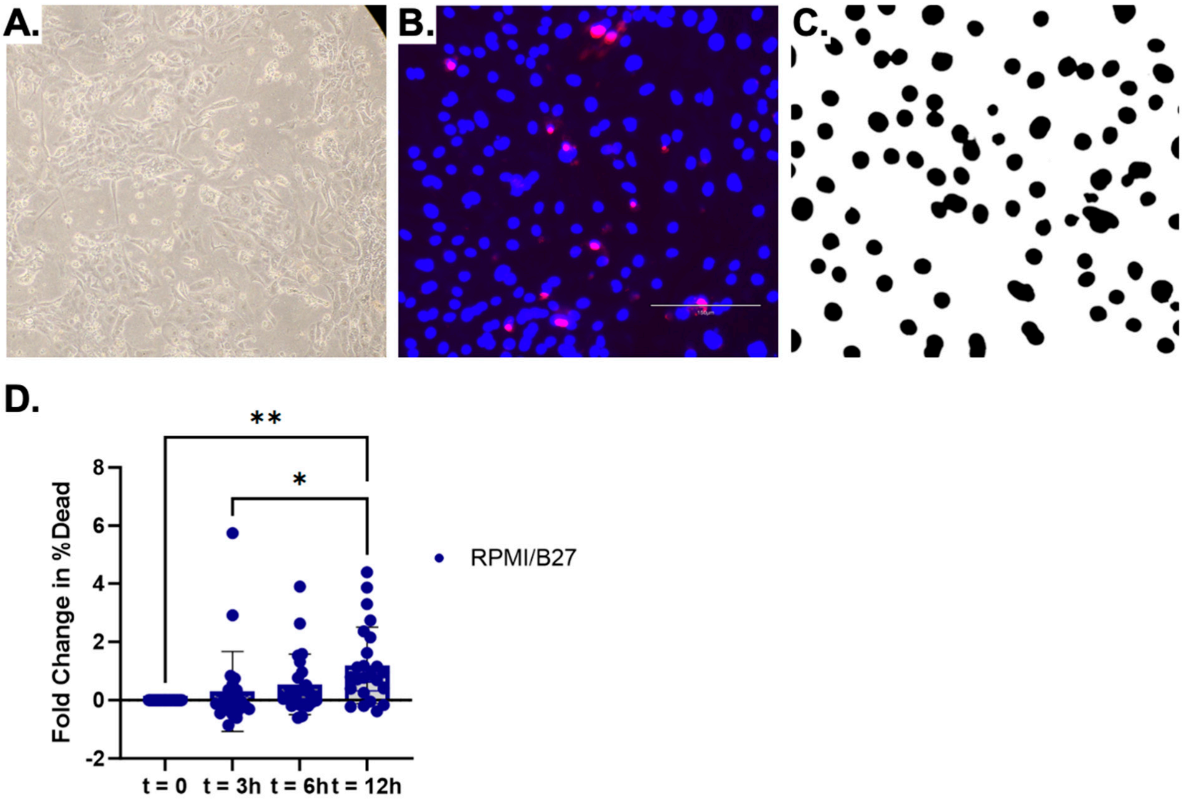


**Supplemental Figure S3. LIVE/DEAD assay for 3-12 hours of hypoxia. (A)** Brightfield image of hiPSC-CMs in 2D culture (10X magnification). **(B)** hiPSC-CMs in hypoxia (1% $O_2$, 5% $CO_2$) at t=3h visualized with LIVE/DEAD stain. DAPI (blue) shows all cell nuclei and RFP (red) shows cell nuclei of dead cells with a compromised plasma membrane integrity. Scale bar is 150 um. **(C)** ImageJ generated binarized image used to count live and dead cells. Binarization was used for two separate images per well: one for live cells and one for dead cells. **(D)** Fold change in percent dead through 12 hours at 1% $O_2$ ($n$ = 24). * $p < 0.05$; ** $p < 0.01$

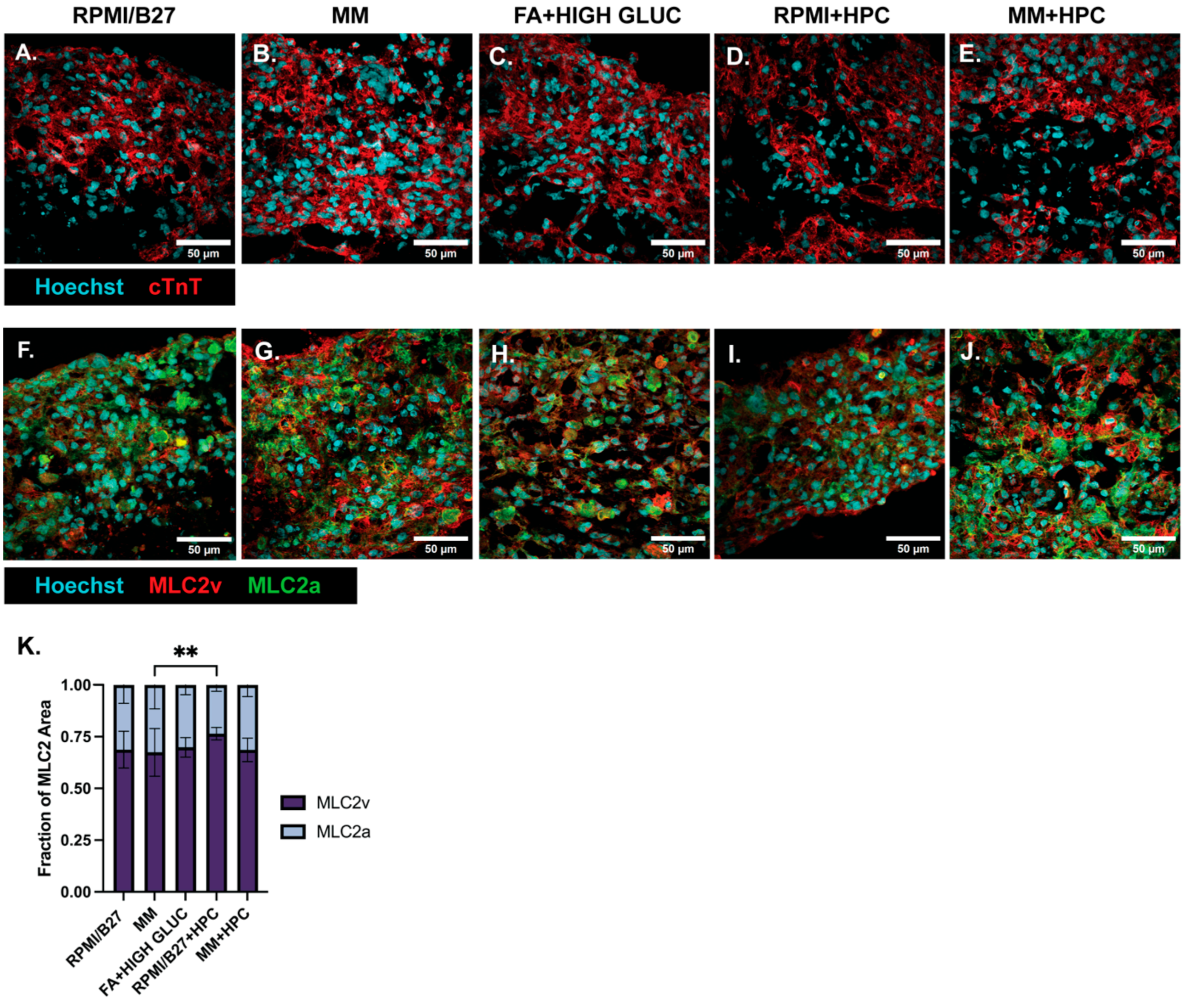


**Supplemental Figure S4. Immunohistochemical staining of ECTs. (A-E)** Histological staining of Hoechst (nuclear stain) and cardiac troponin T, cTnT (sarcomeric stain) of the five treatment conditions after normoxia culture. **(F-J)** Histological staining of myosin light chain 2, specifically MLC2a which represents the immature isoform and MLC2v which represents the more mature isoform as well as Hoechst (nuclear stain) of the five treatment conditions after normoxia culture. **(K)** Quantification of MLC2 isoforms in normoxia ECTs. n = 4-8 samples per group with significance defined as **$p < 0.01$.

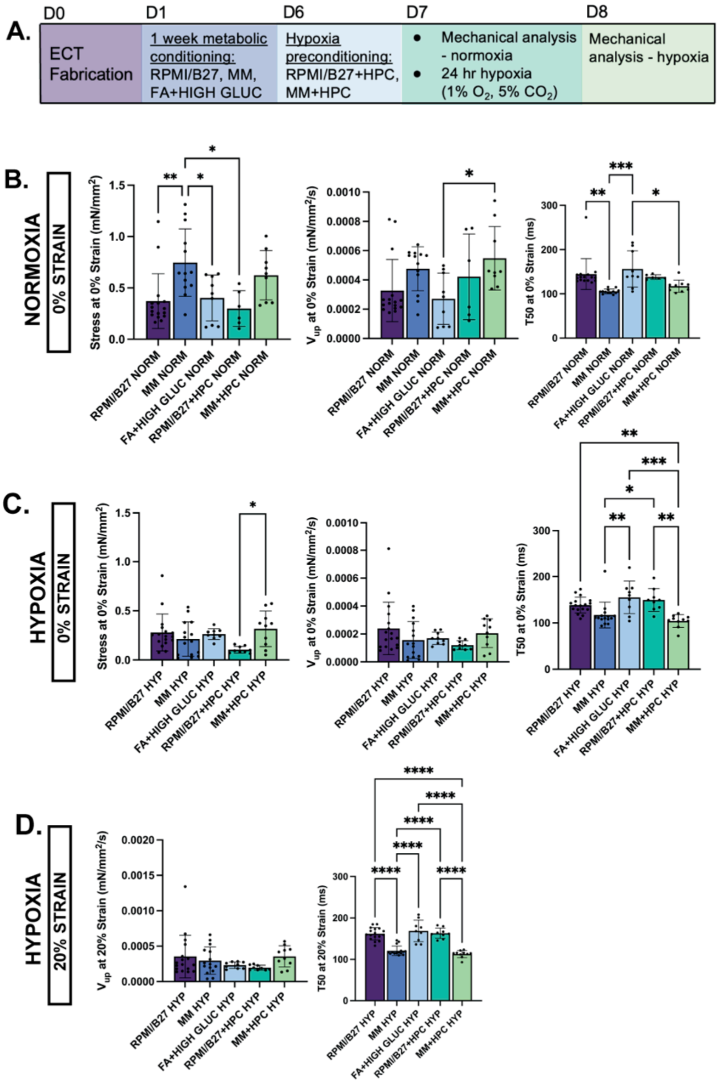


**Supplemental Figure S5. Active stress generation and kinetics of normoxic and hypoxic ECTs. (A)** Timeline for ECTs in normoxia and hypoxia conditions**. (B)** Normoxia ECTs including active stress generation, upstroke Velocity ($V_{up}$) and time to 50% relaxation ($T_{50}$) at 0% strain. **(C)** Hypoxia ECTs including active stress generation, upstroke Velocity (Vup) and time to 50% relaxation (T50) at 0% strain. **(D)** Hypoxia ECTs including upstroke Velocity (Vup) and time to 50% relaxation (T50) at 20% strain. *n* = 6-17 samples per group with significance defined as *$p$ < 0.5; **$p$ < 0.01; ***$p$ < 0.001, ****$p$ < 0.0001

**Supplemental Table S1. Fold change in active stress generation (mN/mm$^2$), upstroke velocity($V_{up}$, mN/mm$^2$/s), and time to 50% relaxation (T50, relaxation) from normoxia to hypoxia.** Higher magnitude, positive values indicate a larger decrease in a given parameter for normoxia to hypoxia. *n* = 2-6 per group.

| | | RPMI/B27 | MM | FA+High Glucose | RPMI/B27+ HPC | MM+HPC |
|---|---|---|---|---|---|---|
| **0% Strain** | **Stress** | 0.2737 ±0.07737 | 0.6893 ±0.1526 | 0.5297 ±0.02458 | 0.4728 ±0.3233 | 0.4698 ±0.1249 |
| | **$V_{up}$** | 0.3699 ±0.06387 | 0.7029 ±0.1221 | 0.4930 ±0.03911 | 0.5345 ±0.2940 | 0.5934 ±0.1072 |
| | **T50** | 0.07976 ±0.09912 | -0.2575 ±0.1894 | 0.05460 ±0.04349 | -0.08680 ±0.02585 | 0.09116 ±0.09489 |
| **20% Strain** | **Stress** | 0.2048 ±0.08910 | 0.6696 ±0.1232 | 0.5138 ±0.02658 | 0.4305 ±0.3396 | 0.4911 ±0.09839 |
| | **$V_{up}$** | 0.3125 ±0.07314 | 0.6844 ±0.1014 | 0.4882 ±0.02929 | 0.4402 ±0.3533 | 0.6112 ±0.09074 |
| | **T50** | -0.001923 ±0.03605 | -0.02692 ±0.04251 | 0.03836 ±0.3397 | -0.008414 ±0.09078 | 0.1323 ±0.1102 |

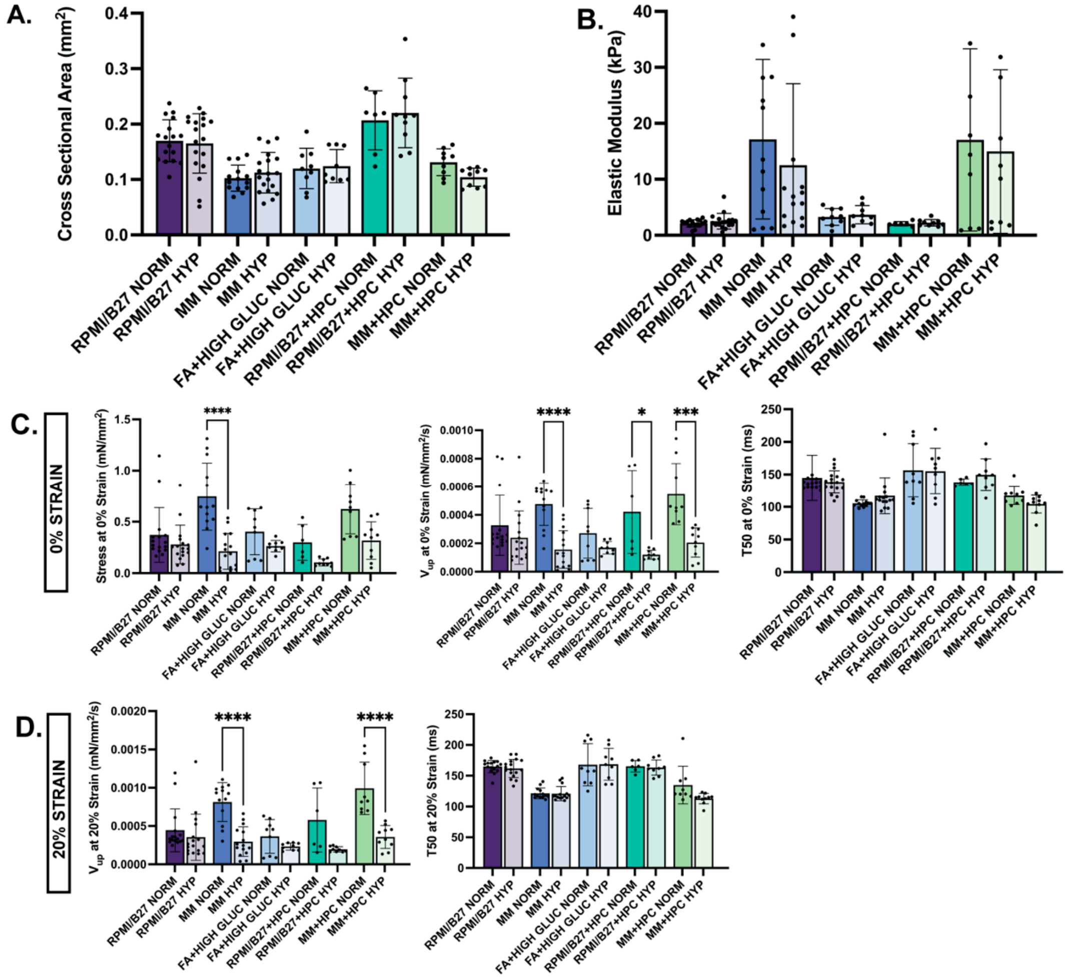


**Supplemental Figure S6. Comparing ECT active stress generation and kinetics after hypoxia treatment.** Comparison of normoxia and hypoxia tissue **(A)** Cross-sectional area (CSA) at D7; **(B)** Elastic Modulus; **(C)** Stress generation, upstroke Velocity ($V_{up}$) and time to 50% relaxation ($T_{50}$) at 0% strain; **(D)** Upstroke velocity ($V_{up}$) and time to 50% relaxation ($T_{50}$) at 20% Strain. $n$ = 6-17 samples per group with significance defined as *$p$ < 0.5; **$p$ < 0.01; ***$p$ < 0.001, ****$p$ < 0.0001

A.

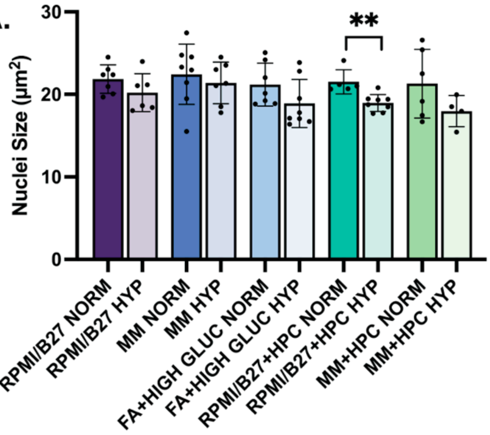


**Supplemental Figure S7. Quantification of nuclei size in normoxic and hypoxic ECTs**. n = 4-8 samples per group with significance defined as $**p < 0.01$.

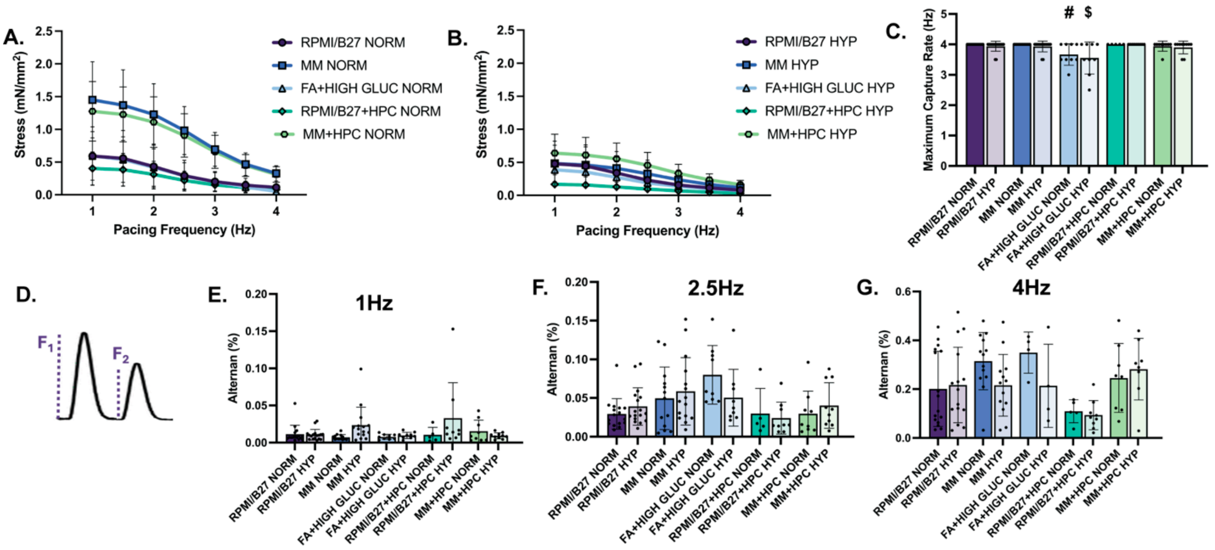


**Supplemental Figure S8. Stress-Frequency response of ECTs in normoxia and hypoxia. (A)** and **(B)** Stress-Frequency plots of normoxia and hypoxia ECTs, respectively. **(C)** Maximum capture rate of ECTs. **(D)** Contractile alternans schematic, illustrating beat-to-beat alterations; **(E)**, **(F)** and **(G)** Quantification of alternans at 1Hz, 2.5Hz and 4Hz, respectively defined as percentage of maximum force ($F_1$-$F_2$/$F_1$). *n* = 5-17 samples per group; # represents significance of at least $p < 0.05$ between FA+high gluc and RPMI/B27 and MM and $ represents significance of at least $p < 0.05$ when compared to all other groups.
